# Self-Medication Practices among a Sample of Latino Migrant Workers in South Florida

**DOI:** 10.3389/fpubh.2014.00108

**Published:** 2014-08-04

**Authors:** Jesús Sánchez

**Affiliations:** ^1^Department of Sociobehavioral and Administrative Pharmacy, Nova Southeastern University, Fort Lauderdale, FL, USA

**Keywords:** self-medication, prevention, Latinos, migrant, US

## Abstract

**Introduction:** Although the literature on self-medication among Latino migrant workers (LMWs) is sparse, a few existing studies indicate that this practice is common in this community. The purpose of this paper is to estimate health status, access to health care, and patterns of self-medication practices of a cohort of LMWs in South Florida.

**Methods:** A stratified network-based sample was utilized to recruit 278 LMWs in the Homestead area. After screening for eligibility, participants were administered a structured questionnaire that collected data on their health status, access to health care services, and self-medication practices. A convenience sample of 24 LMWs, who participated in the parent study were invited back to participate in 3 focus groups to look more in depth into self-medication practices in the LMW community.

**Results:** Study findings indicate that LMWs are affected by a vast array of health problems yet lack access to health care services. Participants already engaged in self-medication practices in the countries of origin and, upon their arrival in the US, these practices continue and, in many cases, increase.

**Conclusion:** Long-held traditions and lack of access to the formal health care system in the US contribute to the high prevalence of self-medication among LMWs. Self-medication practices such as the use of prescription medications without a prescription and lay injection are high risk practices that can have harmful consequences. Prevention interventions that address self-medication in the LMW community are likely to be most effective if they are culturally adapted to the community and facilitate access to health care services.

## Introduction

The concept of self-health management includes behaviors such as health maintenance and illness prevention, symptom evaluation, self-diagnosis, and self-medication (1, 2). Numerous studies have estimated that a significant proportion of illness episodes are handled by some form of self-medication and are not brought to the attention of a health professional. Laypersons do indeed carry on extension medication activities as a regular aspect of their self-care (3–7).

Self-medication is defined by the World Health Organization as the selection and individual use of medicines to alleviate symptoms or cure an illness (8). It is important to realize that self-medication does not only involve the use of over the counter (OTC) drugs. The use of prescription drugs should also be included within the rubric of self-medication if it is based on lay decision making. Finally, self-medication – both OTC and prescription drugs – is often times supplemented by home remedies such as herbal therapies (8). When used safely, medications contribute to better health and to a longer and greater quality of life. However, a vast number of users take medications without being fully informed about the associated risks, contradictions, and adverse effects. Moreover, misuse of medications can interfere with desired treatment and cause harmful reactions.

Self-medication is a growing problem in the US due in part to the increasing use of prescription and OTC medications as well as home remedies (1, 2). A dramatic growth in the number of available medications, limited access to health care, gaining popularity of alternative medicines, and low health literacy have been identified as some key contributing factors (1, 2). Over the last few decades, numerous studies conducted in the US have addressed different aspects of self-medication. For instance, numerous studies have focused on specific groups of drugs such as prescription medications (9–12). Others have targeted specific populations (i.e., college students) (13–15) or stakeholders (i.e., health professionals) (16–18). A comparatively small body of literature has focused on self-medication among immigrant populations in the US. Although the number of studies that have focused on Latino immigrants in the US is still small, there is enough evidence to suggest that self-medication is a common practice among this population. For instance, it is well documented that substantial self-medication with antibiotics obtained without a prescription is prevalent in the Latino immigrant community (19–21). Other studies have directed their attention toward the misuse of prescription medications among Latino immigrants upon their arrival to the US (22–24). A few studies have described home remedies derived from their indigenous medical systems that involve the use of plants and animal-based compounds (25, 26). Finally, some studies have addressed not only those drugs and/or other substances being used as part of self-medication practices but also the routes of administration (i.e., lay injection) used to deliver them (27, 28).

The purpose of this paper is to expand our knowledge of self-medication practices among Latino immigrants in the US by estimating the prevalence of these practices and examining self-medication related behaviors among a cohort of Latino migrant workers (LMWs) in South Florida.

## Materials and Methods

Project Salud – officially entitled “HIV Risk Reduction among High Risk Latino Migrant Workers in South Florida” – was a 4-year study funded by the National Institute on Minority Health and Health Disparities. The main objective of Project Salud was to assess the differential effectiveness of an adapted stage-enhanced motivational interviewing (A-SEMI) intervention compared with a health promotion comparison (HPC) condition for producing reductions in HIV risk and increasing health behaviors among LMWs in the Homestead area of South Florida. The design of the A-SEMI intervention is considered to be an enhancement over existing cognitive behavioral risk reduction approaches because A-SEMI integrates key contextual components from effective HIV prevention interventions (i.e., peer counseling) linked to maintenance of risk reduction effects while culturally adapting these components to the specific needs of the LMW community (29).

Project Salud was conceived and implemented as a community-based participatory research (CBPR) project with the goal of engaging the LMW community in the implementation of an HIV prevention intervention. By framing Project Salud within the CBPR approach, this study responded to the National Institutes of Health priority on establishing equitable partnerships between community members and researchers with the final goal of increasing community participation in the research process, improving community health, and reducing HIV-related health disparities.

A stratified network-based sample of 278 LMWs was recruited from November 2008 to December 2010 from migrant communities in the Homestead area in Miami-Dade County, FL, USA. Homestead is part of a predominantly rural area in the South of Miami-Dade County, FL, USA. Official census data indicate that most of the population in Homestead (51.8%) is Hispanic/Latino, more than one-third (36%) is foreign born, and a majority (57.3%) speaks a language other than English at home (30). Agriculture and nursery constitute an important business in the Homestead area allowing for access to seasonal farm work. Homestead’s LMW population is composed primarily of recently arrived, young, single, or married men that are in the US alone. They live in small crowded apartments with family or friends. Most are Mexican and Central American arriving in this area with little or no English language skills and very limited resources.

After screening for eligibility, participants were administered a structured baseline questionnaire using audio computer-assisted self-interview (A-CASI) with the purpose of enhancing confidentiality among participants as well as increasing comprehension among participants with low literacy. A project staff member was always available during data collection to assist participants with any questions and/or technical difficulties. Each assessment was conducted in Spanish and took approximately 90 min. A project staff member secured the interview data as soon as participants had finished the assessment. The questionnaire collected information on basic sociodemographic information, health status, alcohol, and other drug use (both licit and illicit) history, sexual behaviors, acculturation, and behavioral intentionality. Descriptive statistics were calculated using IBM SPSS Version 21.

Following the baseline assessment, participants were randomly assigned to the A-SEMI or HPC interventions using a computer-generated randomization table. Follow-up assessments were conducted 3 and 9 months after the baseline assessment. Following each assessment and intervention session, participants received a monetary incentive. This study was approved by the IRB of Florida International University.

The data and results presented in this paper were also supported by internal funding provided by Nova Southeastern University via a President’s Faculty Research and Development Grant. After the parent study concluded, a nested qualitative study was conducted between May 2012 and May 2014. A convenience sample of 24 LMWs who participated in the parent study were invited back to participate in the qualitative study. Participants were divided into three focus groups whose goal was to address some key health issues affecting the LMW community that were revealed by the parent study. The ultimate goal was explore these issues (i.e., lay injection practices) from the perspective of the community members in order to gain a better understanding on them and learn how to incorporate these issues into new health promotion and HIV prevention strategies.

Focus group methodology was chosen because of its ability and flexibility to explore new issues (31). Focus groups followed the same procedures and drew upon similar protocol guidelines according to published methods (32, 33). Each focus group was facilitated in Spanish by a project member with experience in focus group facilitation and audio taped. Another project member was present at each session as a note-taker. Project members began by making a 5-min presentation on key health issues that became relevant during the parent study. This initial presentation provided a context for the group from which to proceed. Participants received a monetary incentive for their participation. This study was approved by the IRB of Nova Southeastern University.

Each session was digitally recorded and transcribed. We analyzed data using QSR NVivo 10. Key phrases were identified and each phrase was given a label. Once labeling was completed, similar statements were grouped into themes. A code map was developed to track the data analysis (34). Transcripts and field notes were reviewed by the principal investigator. A second staff member checked the coding and classification system to verify the coding method.

In order to be eligible for the parent study and the subsequent qualitative study, potential participants had to meet the following eligibility criteria: (1) be of Latino origin; (2) 18 years of age or older; (3) have a “farm card”; (4) one or more self-reported episodes of unprotected sex in the past 3 months; (5) willing to be randomized to treatment and contacted for follow-up assessments; (6) likely to be in the general geographic area for 6 months; and (7) able to understand and provide written informed consent.

## Results

### Quantitative results

The sociodemographic characteristics of the 278 study participants are described in Table 1. More than half the sample (54.3%) was male, which does not represent the traditional gender ratio among LMWs in the US whereby males tend to be overrepresented. However, our study aimed for an equal gender distribution. The mean age at interview was 37 and two-thirds of the sample fell between the ages of 31 and 43. As would be expected, almost half the sample (43.2%) was of Mexican origin, followed by Guatemalans (20.1%), and Hondurans (10.1%). The remainder of the sample (26.6%) was comprised of people who came from other countries such as El Salvador, Nicaragua, and Dominican Republic. Slightly more than one-quarter of the sample (27.7%) had no formal education. While slightly more than two-thirds of the sample (68.7%) reported some formal education, only a small minority of them (3.6%) reported at least a high school diploma or GED. On average, study participants had lived 7.5 years in the US and two-thirds of study participants have lived in the US between 3 and 11 years. A large majority of study participants (88.1%) did not have any type of health insurance or primary physician (75.5%). Almost two-thirds of the sample (61.9%) indicated that they did not receive needed medical care in the last 12 months and among those who indicated so, a large majority of them (79.9%) cited lack of health insurance as the main reason.

**Table 1:** **Sociodemographic data among 278 Latino migrant workers**.

Variables	(%)
Gender
Male	54.3
Female	45.7
Age, mean (SD)	37.2 (5.88)
Country of origin
Mexico	43.2
Guatemala	20.1
Honduras	10.1
Others	26.6
Education
No formal education	27.7
<High school degree	68.7
>High school degree	3.6
Years living in the US, mean (SD)	7.5 (4.2)
Health insurance
No	88.1
Yes	11.9
Primary physician
No	75.5
Yes	24.5
Received medical care when needed in last 12 months
No	61.9
Yes	38.1
Main reason why you did not receive medical care
No medical insurance	79.9
I got better	12.9
Other reason	7.2

As shown in Table 2, study participants self-reported a high prevalence of different health issues in the last 12 months prior to interview. A large majority of LMWs in the study reported a myriad of health issues. Dermatitis (84.8%), respiratory condition (73.5%), oral disease (66.3%), infectious disease (60.3%), and eye condition (54.1%) affected more than half the study participants in the last 12 months. Musculoskeletal disorders were less prevalent (45.5%) but still affected a large number of participants. More than one-quarter (28.2%) of the sample experienced alcohol abuse over the last 12 months while 25.9% suffered from depression and 8.3% experienced some other substance abuse disorder over the same time period. Cancer (20.5%) and traumatic injury (14.7%) were less prevalent. Finally, a relatively small percentage (11.4%) of female study participants (*n* = 127) experienced some type of prenatal complication.

**Table 2:** **Self-reported health problems in the last 12 months**.

Health condition	(%)
Dermatitis	84.8
Respiratory condition	73.5
Oral disease	66.3
Infectious disease	60.3
Eye condition	54.1
Musculoskeletal disorder	45.5
Alcohol abuse	28.2
Depression	25.9
Cancer	20.5
Traumatic injury	14.7
Prenatal (*n* = 127)	11.4
Other substance abuse	8.3

We also asked study participants about the health related symptoms they experienced after finishing their day of work in the 30 days prior to interview (Table 3). More than two-thirds of the sample reported a varied range of symptoms that included irritation of the skin (86.8%), irritation of the nose (81.3%), burning sensation in the eyes (77.0%), annoying sensation in the throat (76.2%), coughing and/or breathing problems (73.5%), weakness (68.6%), and headache (62.7%). Almost one-third (31.3%) of study participants reported fainting and/or feeling nausea. A small percentage of participants reported difficulty to speak (9.8%) and convulsions (7.4%).

**Table 3:** **Self-reported health symptoms experienced right after work in the last 30 days**.

Symptoms	(%)
Irritation of the skin	86.8
Irritation of the nose	81.3
Burning sensation in the eyes	77
Annoying sensation in the throat	76.2
Coughing/breathing problems	73.5
Weakness	68.6
Headache	62.7
Fainting/feeling nausea	31.3
Difficulty to speak	9.8
Convulsions	7.4

Table 4 describes the most common OTC, prescription drugs, and herbal remedies used by study participants. The use of OTC medication is widely spread among study participants. In the last 12 months prior to interview, a large majority of the sample reported the use of allergy medication (91.2%), respiratory infection medication (88.6%), other infections medication (77.3%), and pain relief medication (75.2%). More than half of study participants (60.3%) reported the use of digestive disorders medications while less than half of the sample (40.8%) reported using OTC medication to treat fever episodes. Finally, about one-third of the study participants (30.7%) reported the use of vitamins.

**Table 4:** **Self-reported types of medication and home remedies used in the last 12 months**.

Type of medication	(%)
Over the counter
Allergies	91.2
Respiratory infection	88.6
Other infections	77.3
Pain relief	75.2
Digestive disorders	60.3
Fever	40.8
Vitamins	30.7
Prescription
Allergies	76.4
Antibiotics	66.2
Skin medication	55
Analgesics	51.8
Tranquilizers and muscle relaxants	47.7
Rheumatism	33.6
Blood pressure	29.5
Cholesterol	27.7
Antidepressants and stimulants	25.3
Diabetes	18.1
Herbal remedies
Chamomile (digestive problems, menstrual cramps, anxiety, eye infection, and mouth problems)	93.5
Garlic (heart condition and respiratory infection)	90.5
Aloe vera (respiratory infection, digestive problems, and minor injuries)	86.4
Eucalyptus (respiratory infection, allergies, and burns)	83.6
Rue plant (digestive problems, respiratory infection, heart condition, menstrual cramps, rheumatic problems, and bites)	80.2
Peppermint (respiratory infection, allergies, mouth problems, digestive problems, pain)	79.3
Basil (bites, digestive problems, anti-inflammatory, and allergies)	75.7
Lay injection	34.8

Table 4 also reflects the self-reported use of prescription drugs without a prescription. More than three-quarters of the study participants (76.4%) reported using allergy medications. Two-thirds of the sample (66.2%) reported the use of antibiotics. More than half of study participants reported the use of skin medication (55.0%) and analgesics (51.8%) while almost half of them (47.7%) reported the use of tranquilizers and muscle relaxants. One-third of the sample (33.6%) used rheumatism medication over the last 12 months and over one-quarter of the study sample (29.5%) reported the use of blood pressure medication, cholesterol medication (27.7%), and antidepressants and/or stimulants (25.3%). Finally, 18.1% of study participants reported the use of diabetes medication.

Study participants were also asked to self-report their use of herbal remedies and the medical conditions the intended to treat with such remedies. The use of chamomile was widely spread (93.5%) among study participants for the treatment of digestive, menstrual, anxiety, eye, and mouth ailments. Garlic is also used by a large majority of the study participants (90.5%) for the treatment of heart ailments and respiratory infections. The next most utilized (86.4%) herbal remedy is aloe vera, which is used for the treatment of respiratory infections, allergies, and burns. Rue plant is used for the treatment of digestive, respiratory, heart, menstrual, rheumatic, and bite-related ailments by a large majority of the study participants (80.2%). The use of peppermint is also very common (79.3%), and study participants reported using it for the treatment of respiratory, allergy, digestive, mouth, and pain-related ailments. Basil was also reported by a large number of study participants (75.7%) for the treatment of bites, digestive problems, allergies, and as an anti-inflammatory.

Finally, we asked study participants about their lay injection practices for the purpose of medical treatment. Slightly more than one-third of participants (34.8%) reported engaging in this practice over the last 12 months prior to interview.

### Qualitative results

Our analysis identified several themes emerging from the focus groups discussions, which were guided by protocol consisting of seven open-ended questions addressing the following issues: reasons for self-medication use of prescription drugs without a prescription, sources of prescription drugs, and lay injection practices.

Regarding reasons for self-medication, the first theme to emerge was related to participants’ experience engaging in self-medication prior to their arrival to the US. Self-medication is not merely a pharmacological process but a sociocultural one. In deciding how to address a health problem, LMWs draw from their own prior experience as well as the experience of those who constitute their social networks. Participants sought advice from relatives, friends, and lay doctors and healers (hierberos/as, sobadores/as, sanadores/as, and curanderos/as). These are practices they were engaging in before they came to the US and are deeply embedded in their cultural fabric.

I got sick and had the same symptoms as my cousin. He had gone to the doctor and got some medication. It worked for him so, when I came down with the same thing a week later, he gave me his leftover medication.

I’ve been sick like that before. Why should I go to a doctor if I know my sickness goes away with this or that medication?

My grandmother showed me how to use peppermint leaves as a tea for stomach pain. You got to be careful though, because if you have too much, you’ll be belching for a while.

The second theme to emerge out of the discussion about reasons why LMWs engage in self-medication reflects the overwhelming difficulties this community perceives in accessing the US health care system. Participants described their familiarity and comfort with health care practices and providers known to them in their country of origin and felt that the health care system in the US is very complex and expensive for those without health insurance. Moreover, transportation to and from medical facilities and fear of deportation were also cited as barriers for seeking formal treatment.

Medical services are very expensive for us. Even if I can see the doctor for free, I still have to pay for tests and medications. Besides, I’m not working while I’m at the doctor so it costs me more money.

The clinic doctor told me she wanted me to get some tests. I had to pay for it and didn’t have the money, so I didn’t go.

I don’t go anywhere I don’t have to go because I’m scared. Because the *migra* can stop you any time and send you back home.

Another theme identified was sources of prescription medication. Participants in the focus groups admitted that almost any prescription medication is readily available and cited a number of sources: friends and family, tiendas/bodegas (convenience stores), pulgeros (flea markets), and sanadores (lay doctors).

Some study participants – most of them had been in the US for a few years – indicated they had social networks that facilitated easy access to a wide range of prescription drugs. They were able to acquire a substantial number of prescription medicines from Mexico, which they actually prefer to US drugs because they are cheaper, stronger, and come with Spanish instructions.

I know this guy. You cannot go to him in an emergency… if you need something in particular. But with chronic ailments, yes, you can go to him and tell him what you’d like to get from back home and he’ll get it.

Recently arrived study participants seemed to lack those types of networks but were already familiar with alternative ways of getting a hold of any drugs they needed. They referred to Latino markets as the best place to obtain medications.

There is a tienda by [popular restaurant in the area]. The sell Latin food and stuff. They have it [drugs] there for you to buy. I mean, they don’t have it out there on the counter; you have to ask *for* it. You have to ask for it carefully. You have to know whom to ask.

With a lack of affordable options, many participants feel that Latin markets are the best place to obtain medications. Although they may express frustration with the high cost of these drugs – compared to what they paid for them in their countries of origin – they appreciate having an alternative to the formal health care system.

They take advantage of our situation. Back home, I could buy the whole box [of prescription drug] for the same money. Here, I only get half the pills. But it is easier, safer and cheaper than going to the doctor for a person that is without [health] insurance.

Finally, given the parent study’s goal of preventing HIV infection among LMWs, the issue of lay injection was extremely relevant and widely discussed during the focus groups. These discussions revealed that most participants – even those who did not engage in lay injections – believe injectable medications were stronger, more effective, and had a faster onset compared to other routes of administration. Participants referred to lay injection as part of the self-care and self-medication practices that are part of their cultural milieu and engaged in such practices in their countries of origin. There was universal agreement among participants in the focus groups that injectable drugs, syringes, and needles are easily obtained without a prescription at pharmacies or even regular supermarkets in those countries. Here in the US, they can have access to them the same way they have access to prescription drugs, namely, their social networks, markets, and lay injectionists.

The most commonly injected medications are antibiotics – they were mentioned most often by participants – followed by pain killers and vitamins. Female participants also mentioned using hormone injections for birth control.

Penicillin cures anything.

In my experience, injections are far superior to pills. I went to the clinic here because I was so ill. But they never cured me. They gave me these pills and they weren’t working. Finally, I was given some injections by this woman. She said she was a nurse back in [country of origin]. It wasn’t until then that I got cured.

Unlike other routes of drug administration, injections are frequently delivered by another person so the theme of lay injectionists emerged as an important one. Participants agreed that only people with knowledge on how to deliver injections should be trusted. However, as they described these injection practices, the informality of these practices became apparent. Participants described being injected by a pluralistic group of people that included lay doctors, relatives, and/or friends who have been taught how to inject or simply learned how to inject by watching someone else do it.

I give injections. People even want to pay me but I won’t take their money. A doctor taught me how to [give injections]. He would come to the camps with other doctors and nurses and they would teach us… first aid. I even have a certificate.

Finally, one more theme emerged regarding lay injections. Participants indicated that syringes and needles are readily available and are normally supplied along with the drugs. They are for the most part aware of the risk of sharing injection equipment, especially as a risk factor for HIV infection. However, they admitted to a significant amount of sharing as long as it was among close family members. There was little consistency as to the appropriate way of sterilization procedures and, although a few informants indicated that bleach should be used, some others talked about using soap, alcohol, and/or boiling the injection equipment. Many of them also admitted not knowing if the lay injectionist delivering injections was using brand new disposable syringes and needles.

It’s scary to think about it. Yes, there have been times when I received an injection and I couldn’t tell if brand new needles were used. I mean, I didn’t see the person opening it [injection equipment] in front of me. I just trusted them. Now that I think about it, I wouldn’t do it that way.

### Limitations

Certain limitations common to studies based on convenience samples and self-reported behaviors should be considered before conclusions are drawn. This study is not necessarily representative of all LMWs in South Florida because a random sample could not be drawn. We have, however, utilized a stratified network-based sampling approach to recruit participants from a variety of locations and settings in the Homestead area during daytime and evening hours on weekdays and weekends in order to increase the representativeness of the sample. The data presented in this paper are self-reported and subject to recall bias. We utilized calendaring techniques in order to facilitate recall and obtain accurate data.

Our study participants came from many different countries and are not necessarily familiar with drug terminology in English, generic and brand names, and whether they are OTC or prescription drugs in the US. As a result, we used a conservative approach to drug coding and classification. We only included in our quantitative data analysis those drugs that were listed in our questionnaire as OTC or prescription drugs and that were readily identified by potential study participants during pilot testing. During focus groups, we probed participants for detail in order to determine what drugs they were referring to so they could be appropriately classified.

## Discussion

Migrant workers – of which a large majority is of Latino origin – are vital to the economy of the US, especially in the agricultural and nursery segment. Nevertheless, the LMW community constitutes a marginalized and underserved population with many unmet basic needs. Language and cultural barriers, substandard living conditions, poverty, occupational hazards, and discrimination contribute to their health problems and limited access to health care as summarized in Tables 1–3. The health care system in the US is complex, posing a challenge even for the educated American consumer. These issues are compounded for LMWs who arrive to the US. The findings in this study clearly reflect their lack of access to the formal health care system and, consequently, a lack of consistent and affordable quality health care that create the type of environment in which community members feel the need to engage in self-medication as summarized in Table 4.

We complemented the quantitative findings of the parent study with an embedded qualitative study that look more in depth into self-medication practices among LMWs. Focus group discussions were attentive to the specific self-medication practices in the community. Findings revealed that in response to the limited affordable and accessible health care services for this community, many LMWs maintain and, often times, increase self-care and self-medication practices that were part of their cultural customs prior to their arrival in the US. Participants described the practice of obtaining prescription medications and injection equipment without a prescription as widespread. Although most of them realized that these practices are illegal in the US, they justified them as necessary and even ethical. In their view, the availability of an array of medications is beneficial to the community since LMWs have no access to the formal health care system and otherwise would not be able to take care of themselves.

Because of long-held traditions in their country of origin, injectable medications are often preferable to oral administration. Commonly used injectable medications such as antibiotics, painkillers, and vitamins are normally administered by individuals with no medical training. Participants also reported the use of shared syringes to be a common practice, which may contribute to the spread of HIV and other infectious diseases (i.e., hepatitis B). Study participants indicated that they are aware of the risks of HIV infection via injection and stated that they are willing to modify their lay injection practices in order to eliminate such risk.

This study adds to the growing body of literature on immigrant populations in the US and the need to devise effective health promotion interventions to deal with their specific cultural background, socio-environmental conditions, and needs. Limited access to the health care system upon their arrival in the US along with personal customs and traditions make it very likely that LMWs will continue to participate in culturally familiar health care practices, which include self-care and self-medication. It is important to address the issue of self-medication in this population, especially its prevalent use of prescription medications and lay injection. Health literacy campaigns should be conducted in the LMW community to inform community members about the potential harm of using medications that are not approved by the Food and Drug Administration, could be adulterated, had possible degradation of active ingredients, can worsen existing medical conditions, and cause drug resistance. In the case of lay injection, the potential harm extends to the spread of infectious diseases in the community.

A constant and reliable source of health literacy should come from encounters with health care professionals as LMWs access the formal health care system. Unfortunately, most members of this community have limited or no access to the formal system, which actually increases their likelihood of engaging in self-medication practices. Thus, self-medication and access to health care services are inextricably connected. Addressing these problems will require a combined approach that increases access to health services and foments health education within a culturally relevant framework.

## Author Contributions

Dr. Jesús Sánchez was the Principal Investigator on the two grants. Dr. Jesús Sánchez designed the study and supervised it in all its phases. Dr. Jesús Sánchez is also responsible for the data analysis, interpretation, and drafting of the paper.

## Conflict of Interest Statement

The author declares that the research was conducted in the absence of any commercial or financial relationships that could be construed as a potential conflict of interest.

## References

[1] Institute for Safe Medication Practices. Protecting U.S. Citizens from Inappropriate Medication Use. Huntingdon Valley, PA: Institute for Safe Medication Practices (2007).

[2] World Self-Medication Industry. The Story of Self-Care and Self-Medication. Ferney-Voltaire: World Self-Medication Industry (2011).

[3] Kaufman DW Kelly JP Rosenberg L Mitchell AA Recent patterns of medication use in the ambulatory adult population of the United States: the slone survey. JAMA (2002) 287:337–44 10.1001/jama.287.3.337 11790213

[4] Eisenberg D Davis R Ettner SL Trends in alternative medicine use in the U.S., 1990-1997. JAMA (1998) 280:1569–75 10.1001/jama.280.18.1569 9820257

[5] Roper ASW Health Literacy & the Prescription Drug Experience: The Front Line Perspective from Patients, Physicians and Pharmacists (2002). Available from: www.ethnicphysicians.org/publications/Health%20Literacy%20%20the%20Prescription%20Drug%20Experience.pdf

[6] Consumer Reports. The Mainstreaming of Alternative Medicine. (2011). Available from: http://www.consumerreports.org/cro/2012/04/alternative-treatments/index.htm 11010636

[7] Kaiser Family Foundation. Prescription Drug Trends (2007). Available from: http://kff.org/health-costs/fact-sheet/prescription-drug-trends-fact-sheet-may-2010/

[8] World Health Organization. The Role of the Pharmacist in Self-Care and Self-Medication. The Hague: World Health Organization (1998).

[9] McCabe SE Teter CJ Boyd CJ Medical use, illicit use, and diversión of abusable prescription drugs. J Am Coll Health (2006) 54(5):269–78 10.3200/JACH.54.5.269-278 16539219 PMC1876754

[10] Compton WM Volkow ND Abuse of prescription drugs and the risk of addiction. Drug Alcohol Depend (2006) 83(S1):S4–7 10.1016/j.drugalcdep.2005.10.020 16563663

[11] McCabe SE Boyd CJ Teter CJ Subtypes of nonmedical prescription drug misuse. Drug Alcohol Depend (2009) 102:63–70 10.1016/j.drugalcdep.2009.01.007 19278795 PMC2975029

[12] Goldsworthy RC Schwartz NC Mayhorn CB Beyond abuse and exposure: framing the impact of prescription-medication sharing. Am J Public Health (2008) 98(6):1115–21 10.2105/AJPH.2007.123257 18445792 PMC2377306

[13] Prudhomme-White B Becker-Blease KA Grace-Bishop K Stimulant medication use, misuse and abuse in an undergraduate and graduate student sample. J Am Coll Health (2006) 54(5):261–8 10.3200/JACH.54.5.261-268 16539218

[14] Arria AM DuPont RL Nonmedical prescription stimulant use among college students: why we need to do something and what we need to do. J Addict Dis (2010) 29(4):417–26 10.1080/10550887.2010.509273 20924877 PMC2951617

[15] Judson R Langdon SW Illicit use of prescription stimulants among college students: prescription status, motives, theory of planned behavior, knowledge and self-diagnostic tendencies. Psychol Health Med (2009) 14(1):97–104 10.1080/13548500802126723 19085316

[16] Inciardi JA Surrat HL Kurtz SP Burke JJ The diversion of prescription drugs by health care workers in Cincinnati, Ohio. Subst Use Misuse (2006) 41:255–64 10.1080/10826080500391829 16393746

[17] Alison AM Trinkoff RN Storr CL Wall MP Prescription-type drug misuse and workplace access among nurses. J Addict Dis (1999) 18(1):9–17 10.1300/J069v18n01_02 10234559

[18] Baldisseri MR Impaired healthcare professional. Crit Care Med (2007) 35(2):106–16 10.1097/01.CCM.0000252918.87746.96 17242598

[19] Corbett KK Gonzales R Leeman-Castillo BA Flores E Maselli J Kadafar K Appropriate antibiotic use: variation in knowledge and awareness by Hispanic ethnicity and language. Prev Med (2005) 40:162–9 10.1016/j.ypmed.2004.05.016 15533525

[20] Larson EL Dilone J Garcia M Smolowitz J Factors which influence Latino community members to self-prescribe antibiotics. Nurs Res (2006) 55(2):94–102 10.1097/00006199-200603000-00004 16601621

[21] Mainous AG Diaz VA Carnemolla M A community intervention to decrease antibiotics used for self-medication among Latino adults. Ann Fam Med (2009) 7(6):520–6 10.1370/afm.1061 19901311 PMC2775608

[22] Johnson TP VanGeest JB Cho YI Migration and substance use: evidence from the US national health interview survey. Subst Use Misuse (2002) 37(8):941–72 10.1081/JA-120004160 12180572

[23] Horton S Stewart A Reasons for self-medication and perceptions of risk among Mexican American migrant farm workers. J Immigr Minor Health (2012) 14:664–72 10.1007/s10903-011-9562-6 22170398

[24] Arcury TA Quandt SA Delivery of health services to migrant and seasonal farmworkers. Annu Rev Public Health (2007) 28:345–63 10.1146/annurev.publhealth.27.021405.102106 17291182

[25] Waldstein A Popular medicine and self-care in a Mexican migrant community: toward an explanation of an epidemiological paradox. Med Anthropol (2010) 29(1):71–107 10.1080/01459740903517386 20391159

[26] Pylypa J Self-medication practices in two California Mexican communities. J Immigr Health (2001) 3(2):59–75 10.1023/A:1009509815804 16228790

[27] McVea KLSP Lay injection practices among migrant farmworkers in the age of AIDS: evolution of a biomedical folk practice. Soc Sci Med (1997) 45(1):91–8 10.1016/S0277-9536(96)00318-8 9203274

[28] Denner J Organista KC Dupree JD Thrush G Predictors of HIV transmission among migrant and marginally housed Latinos. AIDS Behav (2005) 9(2):201–10 10.1007/s10461-005-3901-3 15933839

[29] Sanchez J Rosa M Serna CA Project Salud: efficacy of a community-based HIV prevention intervention for Hispanic migrant workers in South Florida. AIDS Educ Prev (2013) 25(5):363–75 10.1521/aeap.2013.25.5.363 24059875 PMC3947884

[30] United States Census Bureau. State and County Quick Facts. Homestead, FL: Population Estimates (2000). Available from: http://quickfacts.census.gov/qfd/states/12/1232275.html

[31] Diamond WD Gagnon JP Obtaining pharmacy class feedback through the use of focus group interviews. Am J Pharm Educ (1985) 49:49–54

[32] Krueger RA, editor. Focus Groups: A Practical Guide for Applied Research. 2nd ed Thousand Oaks, CA: Sage (1994).

[33] Morgan DL, editor. Successful Focus Groups: Advancing the State of the Art. Newbury Park, CA: Sage (1993).

[34] Miles MB Huberman AM, editors. Qualitative Data Analysis: An Expanded Sourcebook. 2nd ed Thousand Oaks, CA: Sage (1994).

